# Additional records for *Stenosagola newtoni* Park & Carlton, 2013 (Staphylinidae: Pselaphinae: Faronitae), with notes on aedeagal morphology

**DOI:** 10.3897/BDJ.2.e4135

**Published:** 2014-10-24

**Authors:** Stephen E. Thorpe

**Affiliations:** † School of Biological Sciences (Tamaki Campus), University of Auckland, Auckland, New Zealand

**Keywords:** Hope River Forest Fragmentation Project, New Zealand, *Stenosagola newtoni*

## Abstract

The purpose of this short note is to identify morphospecies sp. 0471 of the Hope River Forest Fragmentation Project. It is *Stenosagola newtoni* Park & Carlton, 2013. The material listed herein raises the number of published specimen records for *Stenosagola newtoni* from 2 to 51. All additional 49 specimens are fully winged males, collected in flight intercept traps (FITs), from the South Island of New Zealand. Examination of the new material has resulted in the detection of an error in the original description of *Stenosagola newtoni*, whereby the mirror image of the aedeagus was inadvertently illustrated. It is not a case of genitalic antisymmetry.

## Introduction

Taxonomic revision of the genus *Stenosagola* Broun, 1921 (Park and Carlton 2013) has facilitated the identification of morphospecies sp. 0471 from the Hope River Forest Fragmentation Project (HRFFP). For an overview of HRFFP, see Ewers et al. (2002). It is a study on the effects of forest fragmentation on native biodiversity in New Zealand. Morphospecies sp. 0471 is herein determined to be *Stenosagola newtoni* Park & Carlton, 2013, hitherto known only from the holotype and a single paratype (both males). 49 additional specimens are herein recorded, all from HRFFP. 42 specimens of sp. 0471 were used in the analysis by Ewers et al. (2007), but one specimen (#0934-008) is herein determined to belong to the Euplectitae. 8 additional specimens from the HRFFP samples, but not used in the analysis, are herein recorded. Morphospecies sp. 0471 was referred to as Euplectini [Euplectitae] sp. 27 in the analysis, but this placement is incorrect (except for specimen #0934-008), as is immediately evident from the paired tarsal claws.

## Materials and methods

24 out of the 49 fully winged HRFFP specimens (~50%) were dissected (or had the aedeagus clearly visible without dissection), including at least one from each site (Bush Hut, Donut, Front Dismal, High Ridge, Home Range, Kakapo, Meat Safe, Prairie, St. James, Twin, Umbrella, Windy Point). The remaining specimens are assumed to be males because fully winged females are unknown in *Stenosagola* (Park and Carlton 2013), and are identified as *Stenosagola newtoni* by association with the dissected material. Photographs of each specimen and its label(s) have been uploaded to the website NatureWatch NZ. The species was identified by comparison of the aedeagus to figures thereof for the genus *Stenosagola* in Park and Carlton (2013). The aedeagi of the HRFFP material was a perfect match for fig. 12d therein, except for being the mirror image! Note that the aedeagus in dorsal view can be recognised by the presence of a small basal lobe, which is absent in ventral view, and by having concave longitudinal curvature. See below for more discussion on this issue. All specimens will be deposited in the Entomology Research Museum, Lincoln University (LUNZ), New Zealand.

## Taxon treatments

### Stenosagola newtoni

Park & Carlton, 2013

#### Materials

- **Type status:** Other material. **Occurrence:** catalogNumber: #284-007; recordedBy: Raphael K. Didham; individualCount: 1; sex: male; lifeStage: adult; **Taxon:** scientificName: Stenosagola newtoni Park & Carlton, 2013; **Location:** country: New Zealand; verbatimLocality: Bush Hut (44.29ha); verbatimLatitude: 42.39S; verbatimLongitude: 172.23E; verbatimCoordinateSystem: degrees minutes; **Identification:** identifiedBy: Stephen E. Thorpe; dateIdentified: 2014; identificationReferences: Park, J.-S.; Carlton, C.E. 2013: A revision of the New Zealand genus Stenosagola Broun, 1921 (Coleoptera: Staphylinidae: Pselaphinae: Faronitae). Coleopterists bulletin, 67(3): 335-359. doi: 10.1649/0010-065X-67.3.335; identificationRemarks: Aedeagus examined. Mirror image of fig. 12d in Park & Carlton (2013); **Event:** eventID: #284; samplingProtocol: FIT; samplingEffort: 16 days; eventDate: 2000-12-18; habitat: forest 128m canopy; **Record Level:** institutionCode: LUNZ; datasetName: Hope River Forest Fragmentation Project; basisOfRecord: PreservedSpecimen
- **Type status:** Other material. **Occurrence:** catalogNumber: #0350-066; recordedBy: Raphael K. Didham; individualCount: 1; sex: male; lifeStage: adult; **Taxon:** scientificName: Stenosagola newtoni Park & Carlton, 2013; **Location:** country: New Zealand; verbatimLocality: Donut (0.69ha); verbatimLatitude: 42.39S; verbatimLongitude: 172.22E; verbatimCoordinateSystem: degrees minutes; **Identification:** identifiedBy: Stephen E. Thorpe; dateIdentified: 2014; identificationReferences: Park, J.-S.; Carlton, C.E. 2013: A revision of the New Zealand genus Stenosagola Broun, 1921 (Coleoptera: Staphylinidae: Pselaphinae: Faronitae). Coleopterists bulletin, 67(3): 335-359. doi: 10.1649/0010-065X-67.3.335; identificationRemarks: Aedeagus not examined; **Event:** eventID: #0350; samplingProtocol: FIT; samplingEffort: 12 days; eventDate: 2000-12-18; habitat: forest 2m ground; **Record Level:** institutionCode: LUNZ; datasetName: Hope River Forest Fragmentation Project; basisOfRecord: PreservedSpecimen
- **Type status:** Other material. **Occurrence:** catalogNumber: #1049[-001]; recordedBy: Raphael K. Didham; individualCount: 1; sex: male; lifeStage: adult; **Taxon:** scientificName: Stenosagola newtoni Park & Carlton, 2013; **Location:** country: New Zealand; verbatimLocality: Donut (0.69ha); verbatimLatitude: 42.39S; verbatimLongitude: 172.22E; verbatimCoordinateSystem: degrees minutes; **Identification:** identifiedBy: Stephen E. Thorpe; dateIdentified: 2014; identificationReferences: Park, J.-S.; Carlton, C.E. 2013: A revision of the New Zealand genus Stenosagola Broun, 1921 (Coleoptera: Staphylinidae: Pselaphinae: Faronitae). Coleopterists bulletin, 67(3): 335-359. doi: 10.1649/0010-065X-67.3.335; identificationRemarks: Aedeagus examined. Mirror image of fig. 12d in Park & Carlton (2013); **Event:** eventID: #1049; samplingProtocol: FIT; samplingEffort: 14 days; eventDate: 2001-01-15; habitat: forest 2m canopy; **Record Level:** institutionCode: LUNZ; datasetName: Hope River Forest Fragmentation Project; basisOfRecord: PreservedSpecimen
- **Type status:** Other material. **Occurrence:** catalogNumber: #0268-007; recordedBy: Raphael K. Didham; individualCount: 1; sex: male; lifeStage: adult; **Taxon:** scientificName: Stenosagola newtoni Park & Carlton, 2013; **Location:** country: New Zealand; verbatimLocality: Front Dismal (71ha); verbatimLatitude: 42.37S; verbatimLongitude: 172.21E; verbatimCoordinateSystem: degrees minutes; **Identification:** identifiedBy: Stephen E. Thorpe; dateIdentified: 2014; identificationReferences: Park, J.-S.; Carlton, C.E. 2013: A revision of the New Zealand genus Stenosagola Broun, 1921 (Coleoptera: Staphylinidae: Pselaphinae: Faronitae). Coleopterists bulletin, 67(3): 335-359. doi: 10.1649/0010-065X-67.3.335; identificationRemarks: Aedeagus not examined; **Event:** eventID: #0268; samplingProtocol: FIT; samplingEffort: 16 days; eventDate: 2000-12-17; habitat: pasture 256m ground; **Record Level:** institutionCode: LUNZ; datasetName: Hope River Forest Fragmentation Project; basisOfRecord: PreservedSpecimen
- **Type status:** Other material. **Occurrence:** catalogNumber: #0243-010; recordedBy: Raphael K. Didham; individualCount: 1; sex: male; lifeStage: adult; **Taxon:** scientificName: Stenosagola newtoni Park & Carlton, 2013; **Location:** country: New Zealand; verbatimLocality: Front Dismal (71ha); verbatimLatitude: 42.37S; verbatimLongitude: 172.21E; verbatimCoordinateSystem: degrees minutes; **Identification:** identifiedBy: Stephen E. Thorpe; dateIdentified: 2014; identificationReferences: Park, J.-S.; Carlton, C.E. 2013: A revision of the New Zealand genus Stenosagola Broun, 1921 (Coleoptera: Staphylinidae: Pselaphinae: Faronitae). Coleopterists bulletin, 67(3): 335-359. doi: 10.1649/0010-065X-67.3.335; identificationRemarks: Aedeagus not examined; **Event:** eventID: #0243; samplingProtocol: FIT; samplingEffort: 16 days; eventDate: 2000-12-17; habitat: forest 0m ground; **Record Level:** institutionCode: LUNZ; datasetName: Hope River Forest Fragmentation Project; basisOfRecord: PreservedSpecimen
- **Type status:** Other material. **Occurrence:** catalogNumber: #1646[-001]; recordedBy: Raphael K. Didham; individualCount: 1; sex: male; lifeStage: adult; **Taxon:** scientificName: Stenosagola newtoni Park & Carlton, 2013; **Location:** country: New Zealand; verbatimLocality: Front Dismal (71ha); verbatimLatitude: 42.37S; verbatimLongitude: 172.21E; verbatimCoordinateSystem: degrees minutes; **Identification:** identifiedBy: Stephen E. Thorpe; dateIdentified: 2014; identificationReferences: Park, J.-S.; Carlton, C.E. 2013: A revision of the New Zealand genus Stenosagola Broun, 1921 (Coleoptera: Staphylinidae: Pselaphinae: Faronitae). Coleopterists bulletin, 67(3): 335-359. doi: 10.1649/0010-065X-67.3.335; identificationRemarks: Aedeagus examined. Mirror image of fig. 12d in Park & Carlton (2013); **Event:** eventID: #1646; samplingProtocol: FIT; samplingEffort: 14 days; eventDate: 2001-02-11; habitat: forest 64m canopy; **Record Level:** institutionCode: LUNZ; datasetName: Hope River Forest Fragmentation Project; basisOfRecord: PreservedSpecimen
- **Type status:** Other material. **Occurrence:** catalogNumber: #1634-014; recordedBy: Raphael K. Didham; individualCount: 1; sex: male; lifeStage: adult; **Taxon:** scientificName: Stenosagola newtoni Park & Carlton, 2013; **Location:** country: New Zealand; verbatimLocality: Front Dismal (71ha); verbatimLatitude: 42.37S; verbatimLongitude: 172.21E; verbatimCoordinateSystem: degrees minutes; **Identification:** identifiedBy: Stephen E. Thorpe; dateIdentified: 2014; identificationReferences: Park, J.-S.; Carlton, C.E. 2013: A revision of the New Zealand genus Stenosagola Broun, 1921 (Coleoptera: Staphylinidae: Pselaphinae: Faronitae). Coleopterists bulletin, 67(3): 335-359. doi: 10.1649/0010-065X-67.3.335; identificationRemarks: Aedeagus not examined; **Event:** eventID: #1634; samplingProtocol: FIT; samplingEffort: 14 days; eventDate: 2001-02-11; habitat: forest 8m ground; **Record Level:** institutionCode: LUNZ; datasetName: Hope River Forest Fragmentation Project; basisOfRecord: PreservedSpecimen
- **Type status:** Other material. **Occurrence:** catalogNumber: #378[-001]; recordedBy: Raphael K. Didham; individualCount: 1; sex: male; lifeStage: adult; **Taxon:** scientificName: Stenosagola newtoni Park & Carlton, 2013; **Location:** country: New Zealand; verbatimLocality: High Ridge (0.06ha); verbatimLatitude: 42.32S; verbatimLongitude: 172.24E; verbatimCoordinateSystem: degrees minutes; **Identification:** identifiedBy: Stephen E. Thorpe; dateIdentified: 2014; identificationReferences: Park, J.-S.; Carlton, C.E. 2013: A revision of the New Zealand genus Stenosagola Broun, 1921 (Coleoptera: Staphylinidae: Pselaphinae: Faronitae). Coleopterists bulletin, 67(3): 335-359. doi: 10.1649/0010-065X-67.3.335; identificationRemarks: Aedeagus examined. Mirror image of fig. 12d in Park & Carlton (2013); **Event:** eventID: #378; samplingProtocol: FIT; samplingEffort: 14 days; eventDate: 2000-12-19; habitat: forest 0m canopy; **Record Level:** institutionCode: LUNZ; datasetName: Hope River Forest Fragmentation Project; basisOfRecord: PreservedSpecimen
- **Type status:** Other material. **Occurrence:** catalogNumber: #0383-019; recordedBy: Raphael K. Didham; individualCount: 1; sex: male; lifeStage: adult; **Taxon:** scientificName: Stenosagola newtoni Park & Carlton, 2013; **Location:** country: New Zealand; verbatimLocality: High Ridge (0.06ha); verbatimLatitude: 42.32S; verbatimLongitude: 172.24E; verbatimCoordinateSystem: degrees minutes; **Identification:** identifiedBy: Stephen E. Thorpe; dateIdentified: 2014; identificationReferences: Park, J.-S.; Carlton, C.E. 2013: A revision of the New Zealand genus Stenosagola Broun, 1921 (Coleoptera: Staphylinidae: Pselaphinae: Faronitae). Coleopterists bulletin, 67(3): 335-359. doi: 10.1649/0010-065X-67.3.335; identificationRemarks: Aedeagus not examined; **Event:** eventID: #0383; samplingProtocol: FIT; samplingEffort: 14 days; eventDate: 2000-12-19; habitat: pasture 4m ground; **Record Level:** institutionCode: LUNZ; datasetName: Hope River Forest Fragmentation Project; basisOfRecord: PreservedSpecimen
- **Type status:** Other material. **Occurrence:** catalogNumber: #0375-055; recordedBy: Raphael K. Didham; individualCount: 1; sex: male; lifeStage: adult; **Taxon:** scientificName: Stenosagola newtoni Park & Carlton, 2013; **Location:** country: New Zealand; verbatimLocality: High Ridge (0.06ha); verbatimLatitude: 42.32S; verbatimLongitude: 172.24E; verbatimCoordinateSystem: degrees minutes; **Identification:** identifiedBy: Stephen E. Thorpe; dateIdentified: 2014; identificationReferences: Park, J.-S.; Carlton, C.E. 2013: A revision of the New Zealand genus Stenosagola Broun, 1921 (Coleoptera: Staphylinidae: Pselaphinae: Faronitae). Coleopterists bulletin, 67(3): 335-359. doi: 10.1649/0010-065X-67.3.335; identificationRemarks: Aedeagus not examined; **Event:** eventID: #0375; samplingProtocol: FIT; samplingEffort: 14 days; eventDate: 2000-12-19; habitat: forest 2m ground; **Record Level:** institutionCode: LUNZ; datasetName: Hope River Forest Fragmentation Project; basisOfRecord: PreservedSpecimen
- **Type status:** Other material. **Occurrence:** catalogNumber: #0375-064; recordedBy: Raphael K. Didham; individualCount: 1; sex: male; lifeStage: adult; **Taxon:** scientificName: Stenosagola newtoni Park & Carlton, 2013; **Location:** country: New Zealand; verbatimLocality: High Ridge (0.06ha); verbatimLatitude: 42.32S; verbatimLongitude: 172.24E; verbatimCoordinateSystem: degrees minutes; **Identification:** identifiedBy: Stephen E. Thorpe; dateIdentified: 2014; identificationReferences: Park, J.-S.; Carlton, C.E. 2013: A revision of the New Zealand genus Stenosagola Broun, 1921 (Coleoptera: Staphylinidae: Pselaphinae: Faronitae). Coleopterists bulletin, 67(3): 335-359. doi: 10.1649/0010-065X-67.3.335; identificationRemarks: Aedeagus not examined; **Event:** eventID: #0375; samplingProtocol: FIT; samplingEffort: 14 days; eventDate: 2000-12-19; habitat: forest 2m ground; **Record Level:** institutionCode: LUNZ; datasetName: Hope River Forest Fragmentation Project; basisOfRecord: PreservedSpecimen
- **Type status:** Other material. **Occurrence:** catalogNumber: #0374-003; recordedBy: Raphael K. Didham; individualCount: 1; sex: male; lifeStage: adult; **Taxon:** scientificName: Stenosagola newtoni Park & Carlton, 2013; **Location:** country: New Zealand; verbatimLocality: High Ridge (0.06ha); verbatimLatitude: 42.32S; verbatimLongitude: 172.24E; verbatimCoordinateSystem: degrees minutes; **Identification:** identifiedBy: Stephen E. Thorpe; dateIdentified: 2014; identificationReferences: Park, J.-S.; Carlton, C.E. 2013: A revision of the New Zealand genus Stenosagola Broun, 1921 (Coleoptera: Staphylinidae: Pselaphinae: Faronitae). Coleopterists bulletin, 67(3): 335-359. doi: 10.1649/0010-065X-67.3.335; identificationRemarks: Aedeagus not examined; **Event:** eventID: #0374; samplingProtocol: FIT; samplingEffort: 14 days; eventDate: 2000-12-19; habitat: forest 0m ground; **Record Level:** institutionCode: LUNZ; datasetName: Hope River Forest Fragmentation Project; basisOfRecord: PreservedSpecimen
- **Type status:** Other material. **Occurrence:** catalogNumber: #0382-034; recordedBy: Raphael K. Didham; individualCount: 1; sex: male; lifeStage: adult; **Taxon:** scientificName: Stenosagola newtoni Park & Carlton, 2013; **Location:** country: New Zealand; verbatimLocality: High Ridge (0.06ha); verbatimLatitude: 42.32S; verbatimLongitude: 172.24E; verbatimCoordinateSystem: degrees minutes; **Identification:** identifiedBy: Stephen E. Thorpe; dateIdentified: 2014; identificationReferences: Park, J.-S.; Carlton, C.E. 2013: A revision of the New Zealand genus Stenosagola Broun, 1921 (Coleoptera: Staphylinidae: Pselaphinae: Faronitae). Coleopterists bulletin, 67(3): 335-359. doi: 10.1649/0010-065X-67.3.335; identificationRemarks: Aedeagus not examined; **Event:** eventID: #0382; samplingProtocol: FIT; samplingEffort: 14 days; eventDate: 2000-12-19; habitat: pasture 2m ground; **Record Level:** institutionCode: LUNZ; datasetName: Hope River Forest Fragmentation Project; basisOfRecord: PreservedSpecimen
- **Type status:** Other material. **Occurrence:** catalogNumber: #0375-072; recordedBy: Raphael K. Didham; individualCount: 1; sex: male; lifeStage: adult; **Taxon:** scientificName: Stenosagola newtoni Park & Carlton, 2013; **Location:** country: New Zealand; verbatimLocality: High Ridge (0.06ha); verbatimLatitude: 42.32S; verbatimLongitude: 172.24E; verbatimCoordinateSystem: degrees minutes; **Identification:** identifiedBy: Stephen E. Thorpe; dateIdentified: 2014; identificationReferences: Park, J.-S.; Carlton, C.E. 2013: A revision of the New Zealand genus Stenosagola Broun, 1921 (Coleoptera: Staphylinidae: Pselaphinae: Faronitae). Coleopterists bulletin, 67(3): 335-359. doi: 10.1649/0010-065X-67.3.335; identificationRemarks: Aedeagus examined. Mirror image of fig. 12d in Park & Carlton (2013); **Event:** eventID: #0375; samplingProtocol: FIT; samplingEffort: 14 days; eventDate: 2000-12-19; habitat: forest 2m ground; **Record Level:** institutionCode: LUNZ; datasetName: Hope River Forest Fragmentation Project; basisOfRecord: PreservedSpecimen
- **Type status:** Other material. **Occurrence:** catalogNumber: #1771-013; recordedBy: Raphael K. Didham; individualCount: 1; sex: male; lifeStage: adult; **Taxon:** scientificName: Stenosagola newtoni Park & Carlton, 2013; **Location:** country: New Zealand; verbatimLocality: High Ridge (0.06ha); verbatimLatitude: 42.32S; verbatimLongitude: 172.24E; verbatimCoordinateSystem: degrees minutes; **Identification:** identifiedBy: Stephen E. Thorpe; dateIdentified: 2014; identificationReferences: Park, J.-S.; Carlton, C.E. 2013: A revision of the New Zealand genus Stenosagola Broun, 1921 (Coleoptera: Staphylinidae: Pselaphinae: Faronitae). Coleopterists bulletin, 67(3): 335-359. doi: 10.1649/0010-065X-67.3.335; identificationRemarks: Aedeagus not examined; **Event:** eventID: #1771; samplingProtocol: FIT; samplingEffort: 14 days; eventDate: 2001-02-13; habitat: pasture 4m ground; **Record Level:** institutionCode: LUNZ; datasetName: Hope River Forest Fragmentation Project; basisOfRecord: PreservedSpecimen
- **Type status:** Other material. **Occurrence:** catalogNumber: #1771-016; recordedBy: Raphael K. Didham; individualCount: 1; sex: male; lifeStage: adult; **Taxon:** scientificName: Stenosagola newtoni Park & Carlton, 2013; **Location:** country: New Zealand; verbatimLocality: High Ridge (0.06ha); verbatimLatitude: 42.32S; verbatimLongitude: 172.24E; verbatimCoordinateSystem: degrees minutes; **Identification:** identifiedBy: Stephen E. Thorpe; dateIdentified: 2014; identificationReferences: Park, J.-S.; Carlton, C.E. 2013: A revision of the New Zealand genus Stenosagola Broun, 1921 (Coleoptera: Staphylinidae: Pselaphinae: Faronitae). Coleopterists bulletin, 67(3): 335-359. doi: 10.1649/0010-065X-67.3.335; identificationRemarks: Aedeagus examined. Mirror image of fig. 12d in Park & Carlton (2013); **Event:** eventID: #1771; samplingProtocol: FIT; samplingEffort: 14 days; eventDate: 2001-02-13; habitat: pasture 4m ground; **Record Level:** institutionCode: LUNZ; datasetName: Hope River Forest Fragmentation Project; basisOfRecord: PreservedSpecimen
- **Type status:** Other material. **Occurrence:** catalogNumber: #0422-019; recordedBy: Raphael K. Didham; individualCount: 1; sex: male; lifeStage: adult; **Taxon:** scientificName: Stenosagola newtoni Park & Carlton, 2013; **Location:** country: New Zealand; verbatimLocality: Home Range (0ha); verbatimLatitude: 42.37S; verbatimLongitude: 172.27E; verbatimCoordinateSystem: degrees minutes; **Identification:** identifiedBy: Stephen E. Thorpe; dateIdentified: 2014; identificationReferences: Park, J.-S.; Carlton, C.E. 2013: A revision of the New Zealand genus Stenosagola Broun, 1921 (Coleoptera: Staphylinidae: Pselaphinae: Faronitae). Coleopterists bulletin, 67(3): 335-359. doi: 10.1649/0010-065X-67.3.335; identificationRemarks: Aedeagus examined. Mirror image of fig. 12d in Park & Carlton (2013); **Event:** eventID: #0422; samplingProtocol: FIT; samplingEffort: 9 days; eventDate: 2000-12-16; habitat: forest 1024m ground; **Record Level:** institutionCode: LUNZ; datasetName: Hope River Forest Fragmentation Project; basisOfRecord: PreservedSpecimen
- **Type status:** Other material. **Occurrence:** catalogNumber: #0204-007; recordedBy: Raphael K. Didham; individualCount: 1; sex: male; lifeStage: adult; **Taxon:** scientificName: Stenosagola newtoni Park & Carlton, 2013; **Location:** country: New Zealand; verbatimLocality: Kakapo (1060ha); verbatimLatitude: 42.39S; verbatimLongitude: 172.21E; verbatimCoordinateSystem: degrees minutes; **Identification:** identifiedBy: Stephen E. Thorpe; dateIdentified: 2014; identificationReferences: Park, J.-S.; Carlton, C.E. 2013: A revision of the New Zealand genus Stenosagola Broun, 1921 (Coleoptera: Staphylinidae: Pselaphinae: Faronitae). Coleopterists bulletin, 67(3): 335-359. doi: 10.1649/0010-065X-67.3.335; identificationRemarks: Aedeagus not examined; **Event:** eventID: #0204; samplingProtocol: FIT; samplingEffort: 14 days; eventDate: 2000-12-20; habitat: pasture 2m ground; **Record Level:** institutionCode: LUNZ; datasetName: Hope River Forest Fragmentation Project; basisOfRecord: PreservedSpecimen
- **Type status:** Other material. **Occurrence:** catalogNumber: #0190-031; recordedBy: Raphael K. Didham; individualCount: 1; sex: male; lifeStage: adult; **Taxon:** scientificName: Stenosagola newtoni Park & Carlton, 2013; **Location:** country: New Zealand; verbatimLocality: Kakapo (1060ha); verbatimLatitude: 42.39S; verbatimLongitude: 172.21E; verbatimCoordinateSystem: degrees minutes; **Identification:** identifiedBy: Stephen E. Thorpe; dateIdentified: 2014; identificationReferences: Park, J.-S.; Carlton, C.E. 2013: A revision of the New Zealand genus Stenosagola Broun, 1921 (Coleoptera: Staphylinidae: Pselaphinae: Faronitae). Coleopterists bulletin, 67(3): 335-359. doi: 10.1649/0010-065X-67.3.335; identificationRemarks: Aedeagus not examined; **Event:** eventID: #0190; samplingProtocol: FIT; samplingEffort: 14 days; eventDate: 2000-12-20; habitat: forest 256m ground; **Record Level:** institutionCode: LUNZ; datasetName: Hope River Forest Fragmentation Project; basisOfRecord: PreservedSpecimen
- **Type status:** Other material. **Occurrence:** catalogNumber: #0182-010; recordedBy: Raphael K. Didham; individualCount: 1; sex: male; lifeStage: adult; **Taxon:** scientificName: Stenosagola newtoni Park & Carlton, 2013; **Location:** country: New Zealand; verbatimLocality: Kakapo (1060ha); verbatimLatitude: 42.39S; verbatimLongitude: 172.21E; verbatimCoordinateSystem: degrees minutes; **Identification:** identifiedBy: Stephen E. Thorpe; dateIdentified: 2014; identificationReferences: Park, J.-S.; Carlton, C.E. 2013: A revision of the New Zealand genus Stenosagola Broun, 1921 (Coleoptera: Staphylinidae: Pselaphinae: Faronitae). Coleopterists bulletin, 67(3): 335-359. doi: 10.1649/0010-065X-67.3.335; identificationRemarks: Aedeagus not examined; **Event:** eventID: #0182; samplingProtocol: FIT; samplingEffort: 14 days; eventDate: 2000-12-20; habitat: forest 0m ground; **Record Level:** institutionCode: LUNZ; datasetName: Hope River Forest Fragmentation Project; basisOfRecord: PreservedSpecimen
- **Type status:** Other material. **Occurrence:** catalogNumber: #0187-001; recordedBy: Raphael K. Didham; individualCount: 1; sex: male; lifeStage: adult; **Taxon:** scientificName: Stenosagola newtoni Park & Carlton, 2013; **Location:** country: New Zealand; verbatimLocality: Kakapo (1060ha); verbatimLatitude: 42.39S; verbatimLongitude: 172.21E; verbatimCoordinateSystem: degrees minutes; **Identification:** identifiedBy: Stephen E. Thorpe; dateIdentified: 2014; identificationReferences: Park, J.-S.; Carlton, C.E. 2013: A revision of the New Zealand genus Stenosagola Broun, 1921 (Coleoptera: Staphylinidae: Pselaphinae: Faronitae). Coleopterists bulletin, 67(3): 335-359. doi: 10.1649/0010-065X-67.3.335; identificationRemarks: Aedeagus not examined; **Event:** eventID: #0187; samplingProtocol: FIT; samplingEffort: 14 days; eventDate: 2000-12-20; habitat: forest 32m ground; **Record Level:** institutionCode: LUNZ; datasetName: Hope River Forest Fragmentation Project; basisOfRecord: PreservedSpecimen
- **Type status:** Other material. **Occurrence:** catalogNumber: #0876-010; recordedBy: Raphael K. Didham; individualCount: 1; sex: male; lifeStage: adult; **Taxon:** scientificName: Stenosagola newtoni Park & Carlton, 2013; **Location:** country: New Zealand; verbatimLocality: Kakapo (1060ha); verbatimLatitude: 42.39S; verbatimLongitude: 172.21E; verbatimCoordinateSystem: degrees minutes; **Identification:** identifiedBy: Stephen E. Thorpe; dateIdentified: 2014; identificationReferences: Park, J.-S.; Carlton, C.E. 2013: A revision of the New Zealand genus Stenosagola Broun, 1921 (Coleoptera: Staphylinidae: Pselaphinae: Faronitae). Coleopterists bulletin, 67(3): 335-359. doi: 10.1649/0010-065X-67.3.335; identificationRemarks: Aedeagus not examined; **Event:** eventID: #0876; samplingProtocol: FIT; samplingEffort: 13 days; eventDate: 2001-01-16; habitat: forest 0m ground; **Record Level:** institutionCode: LUNZ; datasetName: Hope River Forest Fragmentation Project; basisOfRecord: PreservedSpecimen
- **Type status:** Other material. **Occurrence:** catalogNumber: #1579-005; recordedBy: Raphael K. Didham; individualCount: 1; sex: male; lifeStage: adult; **Taxon:** scientificName: Stenosagola newtoni Park & Carlton, 2013; **Location:** country: New Zealand; verbatimLocality: Kakapo (1060ha); verbatimLatitude: 42.39S; verbatimLongitude: 172.21E; verbatimCoordinateSystem: degrees minutes; **Identification:** identifiedBy: Stephen E. Thorpe; dateIdentified: 2014; identificationReferences: Park, J.-S.; Carlton, C.E. 2013: A revision of the New Zealand genus Stenosagola Broun, 1921 (Coleoptera: Staphylinidae: Pselaphinae: Faronitae). Coleopterists bulletin, 67(3): 335-359. doi: 10.1649/0010-065X-67.3.335; identificationRemarks: Aedeagus examined. Mirror image of fig. 12d in Park & Carlton (2013); **Event:** eventID: #1579; samplingProtocol: FIT; samplingEffort: 14 days; eventDate: 2001-02-14; habitat: forest 512m ground; **Record Level:** institutionCode: LUNZ; datasetName: Hope River Forest Fragmentation Project; basisOfRecord: PreservedSpecimen
- **Type status:** Other material. **Occurrence:** catalogNumber: #1570-014; recordedBy: Raphael K. Didham; individualCount: 1; sex: male; lifeStage: adult; **Taxon:** scientificName: Stenosagola newtoni Park & Carlton, 2013; **Location:** country: New Zealand; verbatimLocality: Kakapo (1060ha); verbatimLatitude: 42.39S; verbatimLongitude: 172.21E; verbatimCoordinateSystem: degrees minutes; **Identification:** identifiedBy: Stephen E. Thorpe; dateIdentified: 2014; identificationReferences: Park, J.-S.; Carlton, C.E. 2013: A revision of the New Zealand genus Stenosagola Broun, 1921 (Coleoptera: Staphylinidae: Pselaphinae: Faronitae). Coleopterists bulletin, 67(3): 335-359. doi: 10.1649/0010-065X-67.3.335; identificationRemarks: Aedeagus examined. Mirror image of fig. 12d in Park & Carlton (2013); **Event:** eventID: #1570; samplingProtocol: FIT; samplingEffort: 14 days; eventDate: 2001-02-14; habitat: forest 0m ground; **Record Level:** institutionCode: LUNZ; datasetName: Hope River Forest Fragmentation Project; basisOfRecord: PreservedSpecimen
- **Type status:** Other material. **Occurrence:** catalogNumber: #1578-044; recordedBy: Raphael K. Didham; individualCount: 1; sex: male; lifeStage: adult; **Taxon:** scientificName: Stenosagola newtoni Park & Carlton, 2013; **Location:** country: New Zealand; verbatimLocality: Kakapo (1060ha); verbatimLatitude: 42.39S; verbatimLongitude: 172.21E; verbatimCoordinateSystem: degrees minutes; **Identification:** identifiedBy: Stephen E. Thorpe; dateIdentified: 2014; identificationReferences: Park, J.-S.; Carlton, C.E. 2013: A revision of the New Zealand genus Stenosagola Broun, 1921 (Coleoptera: Staphylinidae: Pselaphinae: Faronitae). Coleopterists bulletin, 67(3): 335-359. doi: 10.1649/0010-065X-67.3.335; identificationRemarks: Aedeagus not examined; **Event:** eventID: #1578; samplingProtocol: FIT; samplingEffort: 14 days; eventDate: 2001-02-14; habitat: forest 256m ground; **Record Level:** institutionCode: LUNZ; datasetName: Hope River Forest Fragmentation Project; basisOfRecord: PreservedSpecimen
- **Type status:** Other material. **Occurrence:** catalogNumber: #1577-002; recordedBy: Raphael K. Didham; individualCount: 1; sex: male; lifeStage: adult; **Taxon:** scientificName: Stenosagola newtoni Park & Carlton, 2013; **Location:** country: New Zealand; verbatimLocality: Kakapo (1060ha); verbatimLatitude: 42.39S; verbatimLongitude: 172.21E; verbatimCoordinateSystem: degrees minutes; **Identification:** identifiedBy: Stephen E. Thorpe; dateIdentified: 2014; identificationReferences: Park, J.-S.; Carlton, C.E. 2013: A revision of the New Zealand genus Stenosagola Broun, 1921 (Coleoptera: Staphylinidae: Pselaphinae: Faronitae). Coleopterists bulletin, 67(3): 335-359. doi: 10.1649/0010-065X-67.3.335; identificationRemarks: Aedeagus examined. Mirror image of fig. 12d in Park & Carlton (2013); **Event:** eventID: #1577; samplingProtocol: FIT; samplingEffort: 14 days; eventDate: 2001-02-14; habitat: forest 128m ground; **Record Level:** institutionCode: LUNZ; datasetName: Hope River Forest Fragmentation Project; basisOfRecord: PreservedSpecimen
- **Type status:** Other material. **Occurrence:** catalogNumber: #0238-009; recordedBy: Raphael K. Didham; individualCount: 1; sex: male; lifeStage: adult; **Taxon:** scientificName: Stenosagola newtoni Park & Carlton, 2013; **Location:** country: New Zealand; verbatimLocality: Meat Safe (372.74ha); verbatimLatitude: 42.32S; verbatimLongitude: 172.24E; verbatimCoordinateSystem: degrees minutes; **Identification:** identifiedBy: Stephen E. Thorpe; dateIdentified: 2014; identificationReferences: Park, J.-S.; Carlton, C.E. 2013: A revision of the New Zealand genus Stenosagola Broun, 1921 (Coleoptera: Staphylinidae: Pselaphinae: Faronitae). Coleopterists bulletin, 67(3): 335-359. doi: 10.1649/0010-065X-67.3.335; identificationRemarks: Aedeagus examined. Mirror image of fig. 12d in Park & Carlton (2013); **Event:** eventID: #0238; samplingProtocol: FIT; samplingEffort: 17 days; eventDate: 2000-12-15; habitat: pasture 32m ground; **Record Level:** institutionCode: LUNZ; datasetName: Hope River Forest Fragmentation Project; basisOfRecord: PreservedSpecimen
- **Type status:** Other material. **Occurrence:** catalogNumber: #0214-158; recordedBy: Raphael K. Didham; individualCount: 1; sex: male; lifeStage: adult; **Taxon:** scientificName: Stenosagola newtoni Park & Carlton, 2013; **Location:** country: New Zealand; verbatimLocality: Meat Safe (372.74ha); verbatimLatitude: 42.32S; verbatimLongitude: 172.24E; verbatimCoordinateSystem: degrees minutes; **Identification:** identifiedBy: Stephen E. Thorpe; dateIdentified: 2014; identificationReferences: Park, J.-S.; Carlton, C.E. 2013: A revision of the New Zealand genus Stenosagola Broun, 1921 (Coleoptera: Staphylinidae: Pselaphinae: Faronitae). Coleopterists bulletin, 67(3): 335-359. doi: 10.1649/0010-065X-67.3.335; identificationRemarks: Aedeagus examined. Possibly damaged or aberrant, but still closest to S. newtoni; **Event:** eventID: #0214; samplingProtocol: FIT; samplingEffort: 17 days; eventDate: 2000-12-15; habitat: forest 0m ground; **Record Level:** institutionCode: LUNZ; datasetName: Hope River Forest Fragmentation Project; basisOfRecord: PreservedSpecimen
- **Type status:** Other material. **Occurrence:** catalogNumber: #0214-108; recordedBy: Raphael K. Didham; individualCount: 1; sex: male; lifeStage: adult; **Taxon:** scientificName: Stenosagola newtoni Park & Carlton, 2013; **Location:** country: New Zealand; verbatimLocality: Meat Safe (372.74ha); verbatimLatitude: 42.32S; verbatimLongitude: 172.24E; verbatimCoordinateSystem: degrees minutes; **Identification:** identifiedBy: Stephen E. Thorpe; dateIdentified: 2014; identificationReferences: Park, J.-S.; Carlton, C.E. 2013: A revision of the New Zealand genus Stenosagola Broun, 1921 (Coleoptera: Staphylinidae: Pselaphinae: Faronitae). Coleopterists bulletin, 67(3): 335-359. doi: 10.1649/0010-065X-67.3.335; identificationRemarks: Aedeagus examined. Mirror image of fig. 12d in Park & Carlton (2013); **Event:** eventID: #0214; samplingProtocol: FIT; samplingEffort: 17 days; eventDate: 2000-12-15; habitat: forest 0m ground; **Record Level:** institutionCode: LUNZ; datasetName: Hope River Forest Fragmentation Project; basisOfRecord: PreservedSpecimen
- **Type status:** Other material. **Occurrence:** catalogNumber: #0583-001; recordedBy: Raphael K. Didham; individualCount: 1; sex: male; lifeStage: adult; **Taxon:** scientificName: Stenosagola newtoni Park & Carlton, 2013; **Location:** country: New Zealand; verbatimLocality: Meat Safe (372.74ha); verbatimLatitude: 42.32S; verbatimLongitude: 172.24E; verbatimCoordinateSystem: degrees minutes; **Identification:** identifiedBy: Stephen E. Thorpe; dateIdentified: 2014; identificationReferences: Park, J.-S.; Carlton, C.E. 2013: A revision of the New Zealand genus Stenosagola Broun, 1921 (Coleoptera: Staphylinidae: Pselaphinae: Faronitae). Coleopterists bulletin, 67(3): 335-359. doi: 10.1649/0010-065X-67.3.335; identificationRemarks: Aedeagus not examined; **Event:** eventID: #0583; samplingProtocol: FIT; samplingEffort: 14 days; eventDate: 2000-12-29; habitat: pasture 8m ground; **Record Level:** institutionCode: LUNZ; datasetName: Hope River Forest Fragmentation Project; basisOfRecord: PreservedSpecimen
- **Type status:** Other material. **Occurrence:** catalogNumber: #0931-001; recordedBy: Raphael K. Didham; individualCount: 1; sex: male; lifeStage: adult; **Taxon:** scientificName: Stenosagola newtoni Park & Carlton, 2013; **Location:** country: New Zealand; verbatimLocality: Meat Safe (372.74ha); verbatimLatitude: 42.32S; verbatimLongitude: 172.24E; verbatimCoordinateSystem: degrees minutes; **Identification:** identifiedBy: Stephen E. Thorpe; dateIdentified: 2014; identificationReferences: Park, J.-S.; Carlton, C.E. 2013: A revision of the New Zealand genus Stenosagola Broun, 1921 (Coleoptera: Staphylinidae: Pselaphinae: Faronitae). Coleopterists bulletin, 67(3): 335-359. doi: 10.1649/0010-065X-67.3.335; identificationRemarks: Aedeagus examined. Mirror image of fig. 12d in Park & Carlton (2013); **Event:** eventID: #0931; samplingProtocol: FIT; samplingEffort: 14 days; eventDate: 2001-01-12; habitat: pasture 16m ground; **Record Level:** institutionCode: LUNZ; datasetName: Hope River Forest Fragmentation Project; basisOfRecord: PreservedSpecimen
- **Type status:** Other material. **Occurrence:** catalogNumber: #0908-067; recordedBy: Raphael K. Didham; individualCount: 1; sex: male; lifeStage: adult; **Taxon:** scientificName: Stenosagola newtoni Park & Carlton, 2013; **Location:** country: New Zealand; verbatimLocality: Meat Safe (372.74ha); verbatimLatitude: 42.32S; verbatimLongitude: 172.24E; verbatimCoordinateSystem: degrees minutes; **Identification:** identifiedBy: Stephen E. Thorpe; dateIdentified: 2014; identificationReferences: Park, J.-S.; Carlton, C.E. 2013: A revision of the New Zealand genus Stenosagola Broun, 1921 (Coleoptera: Staphylinidae: Pselaphinae: Faronitae). Coleopterists bulletin, 67(3): 335-359. doi: 10.1649/0010-065X-67.3.335; identificationRemarks: Aedeagus examined. Mirror image of fig. 12d in Park & Carlton (2013); **Event:** eventID: #0908; samplingProtocol: FIT; samplingEffort: 14 days; eventDate: 2001-01-12; habitat: forest 0m ground; **Record Level:** institutionCode: LUNZ; datasetName: Hope River Forest Fragmentation Project; basisOfRecord: PreservedSpecimen
- **Type status:** Other material. **Occurrence:** catalogNumber: #0929-013; recordedBy: Raphael K. Didham; individualCount: 1; sex: male; lifeStage: adult; **Taxon:** scientificName: Stenosagola newtoni Park & Carlton, 2013; **Location:** country: New Zealand; verbatimLocality: Meat Safe (372.74ha); verbatimLatitude: 42.32S; verbatimLongitude: 172.24E; verbatimCoordinateSystem: degrees minutes; **Identification:** identifiedBy: Stephen E. Thorpe; dateIdentified: 2014; identificationReferences: Park, J.-S.; Carlton, C.E. 2013: A revision of the New Zealand genus Stenosagola Broun, 1921 (Coleoptera: Staphylinidae: Pselaphinae: Faronitae). Coleopterists bulletin, 67(3): 335-359. doi: 10.1649/0010-065X-67.3.335; identificationRemarks: Aedeagus not examined; **Event:** eventID: #0929; samplingProtocol: FIT; samplingEffort: 14 days; eventDate: 2001-01-12; habitat: pasture 4m ground; **Record Level:** institutionCode: LUNZ; datasetName: Hope River Forest Fragmentation Project; basisOfRecord: PreservedSpecimen
- **Type status:** Other material. **Occurrence:** catalogNumber: #1610-019; recordedBy: Raphael K. Didham; individualCount: 1; sex: male; lifeStage: adult; **Taxon:** scientificName: Stenosagola newtoni Park & Carlton, 2013; **Location:** country: New Zealand; verbatimLocality: Meat Safe (372.74ha); verbatimLatitude: 42.32S; verbatimLongitude: 172.24E; verbatimCoordinateSystem: degrees minutes; **Identification:** identifiedBy: Stephen E. Thorpe; dateIdentified: 2014; identificationReferences: Park, J.-S.; Carlton, C.E. 2013: A revision of the New Zealand genus Stenosagola Broun, 1921 (Coleoptera: Staphylinidae: Pselaphinae: Faronitae). Coleopterists bulletin, 67(3): 335-359. doi: 10.1649/0010-065X-67.3.335; identificationRemarks: Aedeagus not examined; **Event:** eventID: #1610; samplingProtocol: FIT; samplingEffort: 14 days; eventDate: 2001-02-09; habitat: forest 256m ground; **Record Level:** institutionCode: LUNZ; datasetName: Hope River Forest Fragmentation Project; basisOfRecord: PreservedSpecimen
- **Type status:** Other material. **Occurrence:** catalogNumber: #1622-010; recordedBy: Raphael K. Didham; individualCount: 1; sex: male; lifeStage: adult; **Taxon:** scientificName: Stenosagola newtoni Park & Carlton, 2013; **Location:** country: New Zealand; verbatimLocality: Meat Safe (372.74ha); verbatimLatitude: 42.32S; verbatimLongitude: 172.24E; verbatimCoordinateSystem: degrees minutes; **Identification:** identifiedBy: Stephen E. Thorpe; dateIdentified: 2014; identificationReferences: Park, J.-S.; Carlton, C.E. 2013: A revision of the New Zealand genus Stenosagola Broun, 1921 (Coleoptera: Staphylinidae: Pselaphinae: Faronitae). Coleopterists bulletin, 67(3): 335-359. doi: 10.1649/0010-065X-67.3.335; identificationRemarks: Aedeagus not examined; **Event:** eventID: #1622; samplingProtocol: FIT; samplingEffort: 14 days; eventDate: 2001-02-09; habitat: pasture 2m ground; **Record Level:** institutionCode: LUNZ; datasetName: Hope River Forest Fragmentation Project; basisOfRecord: PreservedSpecimen
- **Type status:** Other material. **Occurrence:** catalogNumber: #1625-001; recordedBy: Raphael K. Didham; individualCount: 1; sex: male; lifeStage: adult; **Taxon:** scientificName: Stenosagola newtoni Park & Carlton, 2013; **Location:** country: New Zealand; verbatimLocality: Meat Safe (372.74ha); verbatimLatitude: 42.32S; verbatimLongitude: 172.24E; verbatimCoordinateSystem: degrees minutes; **Identification:** identifiedBy: Stephen E. Thorpe; dateIdentified: 2014; identificationReferences: Park, J.-S.; Carlton, C.E. 2013: A revision of the New Zealand genus Stenosagola Broun, 1921 (Coleoptera: Staphylinidae: Pselaphinae: Faronitae). Coleopterists bulletin, 67(3): 335-359. doi: 10.1649/0010-065X-67.3.335; identificationRemarks: Aedeagus examined. Mirror image of fig. 12d in Park & Carlton (2013); **Event:** eventID: #1625; samplingProtocol: FIT; samplingEffort: 14 days; eventDate: 2001-02-09; habitat: pasture 16m ground; **Record Level:** institutionCode: LUNZ; datasetName: Hope River Forest Fragmentation Project; basisOfRecord: PreservedSpecimen
- **Type status:** Other material. **Occurrence:** catalogNumber: #1628-001; recordedBy: Raphael K. Didham; individualCount: 1; sex: male; lifeStage: adult; **Taxon:** scientificName: Stenosagola newtoni Park & Carlton, 2013; **Location:** country: New Zealand; verbatimLocality: Meat Safe (372.74ha); verbatimLatitude: 42.32S; verbatimLongitude: 172.24E; verbatimCoordinateSystem: degrees minutes; **Identification:** identifiedBy: Stephen E. Thorpe; dateIdentified: 2014; identificationReferences: Park, J.-S.; Carlton, C.E. 2013: A revision of the New Zealand genus Stenosagola Broun, 1921 (Coleoptera: Staphylinidae: Pselaphinae: Faronitae). Coleopterists bulletin, 67(3): 335-359. doi: 10.1649/0010-065X-67.3.335; identificationRemarks: Aedeagus examined. Mirror image of fig. 12d in Park & Carlton (2013); **Event:** eventID: #1628; samplingProtocol: FIT; samplingEffort: 14 days; eventDate: 2001-02-09; habitat: pasture 128m ground; **Record Level:** institutionCode: LUNZ; datasetName: Hope River Forest Fragmentation Project; basisOfRecord: PreservedSpecimen
- **Type status:** Other material. **Occurrence:** catalogNumber: #1629-001; recordedBy: Raphael K. Didham; individualCount: 1; sex: male; lifeStage: adult; **Taxon:** scientificName: Stenosagola newtoni Park & Carlton, 2013; **Location:** country: New Zealand; verbatimLocality: Meat Safe (372.74ha); verbatimLatitude: 42.32S; verbatimLongitude: 172.24E; verbatimCoordinateSystem: degrees minutes; **Identification:** identifiedBy: Stephen E. Thorpe; dateIdentified: 2014; identificationReferences: Park, J.-S.; Carlton, C.E. 2013: A revision of the New Zealand genus Stenosagola Broun, 1921 (Coleoptera: Staphylinidae: Pselaphinae: Faronitae). Coleopterists bulletin, 67(3): 335-359. doi: 10.1649/0010-065X-67.3.335; identificationRemarks: Aedeagus not examined; **Event:** eventID: #1629; samplingProtocol: FIT; samplingEffort: 14 days; eventDate: 2001-02-13; habitat: pasture 256m ground; **Record Level:** institutionCode: LUNZ; datasetName: Hope River Forest Fragmentation Project; basisOfRecord: PreservedSpecimen
- **Type status:** Other material. **Occurrence:** catalogNumber: #0330-001; recordedBy: Raphael K. Didham; individualCount: 1; sex: male; lifeStage: adult; **Taxon:** scientificName: Stenosagola newtoni Park & Carlton, 2013; **Location:** country: New Zealand; verbatimLocality: Prairie (2.88ha); verbatimLatitude: 42.39S; verbatimLongitude: 172.21E; verbatimCoordinateSystem: degrees minutes; **Identification:** identifiedBy: Stephen E. Thorpe; dateIdentified: 2014; identificationReferences: Park, J.-S.; Carlton, C.E. 2013: A revision of the New Zealand genus Stenosagola Broun, 1921 (Coleoptera: Staphylinidae: Pselaphinae: Faronitae). Coleopterists bulletin, 67(3): 335-359. doi: 10.1649/0010-065X-67.3.335; identificationRemarks: Aedeagus examined. Mirror image of fig. 12d in Park & Carlton (2013); **Event:** eventID: #0330; samplingProtocol: FIT; samplingEffort: 15 days; eventDate: 2000-12-18; habitat: pasture 32m ground; **Record Level:** institutionCode: LUNZ; datasetName: Hope River Forest Fragmentation Project; basisOfRecord: PreservedSpecimen
- **Type status:** Other material. **Occurrence:** catalogNumber: #0318-102; recordedBy: Raphael K. Didham; individualCount: 1; sex: male; lifeStage: adult; **Taxon:** scientificName: Stenosagola newtoni Park & Carlton, 2013; **Location:** country: New Zealand; verbatimLocality: Prairie (2.88ha); verbatimLatitude: 42.39S; verbatimLongitude: 172.21E; verbatimCoordinateSystem: degrees minutes; **Identification:** identifiedBy: Stephen E. Thorpe; dateIdentified: 2014; identificationReferences: Park, J.-S.; Carlton, C.E. 2013: A revision of the New Zealand genus Stenosagola Broun, 1921 (Coleoptera: Staphylinidae: Pselaphinae: Faronitae). Coleopterists bulletin, 67(3): 335-359. doi: 10.1649/0010-065X-67.3.335; identificationRemarks: Aedeagus examined. Mirror image of fig. 12d in Park & Carlton (2013); **Event:** eventID: #0318; samplingProtocol: FIT; samplingEffort: 15 days; eventDate: 2000-12-18; habitat: forest 64m ground; **Record Level:** institutionCode: LUNZ; datasetName: Hope River Forest Fragmentation Project; basisOfRecord: PreservedSpecimen
- **Type status:** Other material. **Occurrence:** catalogNumber: #1021-011; recordedBy: Raphael K. Didham; individualCount: 1; sex: male; lifeStage: adult; **Taxon:** scientificName: Stenosagola newtoni Park & Carlton, 2013; **Location:** country: New Zealand; verbatimLocality: Prairie (2.88ha); verbatimLatitude: 42.39S; verbatimLongitude: 172.21E; verbatimCoordinateSystem: degrees minutes; **Identification:** identifiedBy: Stephen E. Thorpe; dateIdentified: 2014; identificationReferences: Park, J.-S.; Carlton, C.E. 2013: A revision of the New Zealand genus Stenosagola Broun, 1921 (Coleoptera: Staphylinidae: Pselaphinae: Faronitae). Coleopterists bulletin, 67(3): 335-359. doi: 10.1649/0010-065X-67.3.335; identificationRemarks: Aedeagus examined. Mirror image of fig. 12d in Park & Carlton (2013); **Event:** eventID: #1021; samplingProtocol: FIT; samplingEffort: 14 days; eventDate: 2001-01-15; habitat: pasture 4m ground; **Record Level:** institutionCode: LUNZ; datasetName: Hope River Forest Fragmentation Project; basisOfRecord: PreservedSpecimen
- **Type status:** Other material. **Occurrence:** catalogNumber: #1703-005; recordedBy: Raphael K. Didham; individualCount: 1; sex: male; lifeStage: adult; **Taxon:** scientificName: Stenosagola newtoni Park & Carlton, 2013; **Location:** country: New Zealand; verbatimLocality: Prairie (2.88ha); verbatimLatitude: 42.39S; verbatimLongitude: 172.21E; verbatimCoordinateSystem: degrees minutes; **Identification:** identifiedBy: Stephen E. Thorpe; dateIdentified: 2014; identificationReferences: Park, J.-S.; Carlton, C.E. 2013: A revision of the New Zealand genus Stenosagola Broun, 1921 (Coleoptera: Staphylinidae: Pselaphinae: Faronitae). Coleopterists bulletin, 67(3): 335-359. doi: 10.1649/0010-065X-67.3.335; identificationRemarks: Aedeagus not examined; **Event:** eventID: #1703; samplingProtocol: FIT; samplingEffort: 14 days; eventDate: 2001-02-12; habitat: forest 8m ground; **Record Level:** institutionCode: LUNZ; datasetName: Hope River Forest Fragmentation Project; basisOfRecord: PreservedSpecimen
- **Type status:** Other material. **Occurrence:** catalogNumber: #482[-001]; recordedBy: Raphael K. Didham; individualCount: 1; sex: male; lifeStage: adult; **Taxon:** scientificName: Stenosagola newtoni Park & Carlton, 2013; **Location:** country: New Zealand; verbatimLocality: St. James (1060407ha); verbatimLatitude: 42.30S; verbatimLongitude: 172.28E; verbatimCoordinateSystem: degrees minutes; **Identification:** identifiedBy: Stephen E. Thorpe; dateIdentified: 2014; identificationReferences: Park, J.-S.; Carlton, C.E. 2013: A revision of the New Zealand genus Stenosagola Broun, 1921 (Coleoptera: Staphylinidae: Pselaphinae: Faronitae). Coleopterists bulletin, 67(3): 335-359. doi: 10.1649/0010-065X-67.3.335; identificationRemarks: Aedeagus examined. Mirror image of fig. 12d in Park & Carlton (2013); **Event:** eventID: #482; samplingProtocol: FIT; samplingEffort: 14 days; eventDate: 2001-01-02; habitat: forest 64m canopy; **Record Level:** institutionCode: LUNZ; datasetName: Hope River Forest Fragmentation Project; basisOfRecord: PreservedSpecimen
- **Type status:** Other material. **Occurrence:** catalogNumber: #1530-001; recordedBy: Raphael K. Didham; individualCount: 1; sex: male; lifeStage: adult; **Taxon:** scientificName: Stenosagola newtoni Park & Carlton, 2013; **Location:** country: New Zealand; verbatimLocality: St. James (1060407ha); verbatimLatitude: 42.30S; verbatimLongitude: 172.28E; verbatimCoordinateSystem: degrees minutes; **Identification:** identifiedBy: Stephen E. Thorpe; dateIdentified: 2014; identificationReferences: Park, J.-S.; Carlton, C.E. 2013: A revision of the New Zealand genus Stenosagola Broun, 1921 (Coleoptera: Staphylinidae: Pselaphinae: Faronitae). Coleopterists bulletin, 67(3): 335-359. doi: 10.1649/0010-065X-67.3.335; identificationRemarks: Aedeagus not examined; **Event:** eventID: #1530; samplingProtocol: FIT; samplingEffort: 14 days; eventDate: 2001-02-13; habitat: pasture 8m ground; **Record Level:** institutionCode: LUNZ; datasetName: Hope River Forest Fragmentation Project; basisOfRecord: PreservedSpecimen
- **Type status:** Other material. **Occurrence:** catalogNumber: #339[-001]; recordedBy: Raphael K. Didham; individualCount: 1; sex: male; lifeStage: adult; **Taxon:** scientificName: Stenosagola newtoni Park & Carlton, 2013; **Location:** country: New Zealand; verbatimLocality: Twin (0.92ha); verbatimLatitude: 42.39S; verbatimLongitude: 172.22E; verbatimCoordinateSystem: degrees minutes; **Identification:** identifiedBy: Stephen E. Thorpe; dateIdentified: 2014; identificationReferences: Park, J.-S.; Carlton, C.E. 2013: A revision of the New Zealand genus Stenosagola Broun, 1921 (Coleoptera: Staphylinidae: Pselaphinae: Faronitae). Coleopterists bulletin, 67(3): 335-359. doi: 10.1649/0010-065X-67.3.335; identificationRemarks: Aedeagus examined. Mirror image of fig. 12d in Park & Carlton (2013); **Event:** eventID: #339; samplingProtocol: FIT; samplingEffort: 14 days; eventDate: 2000-12-18; habitat: forest 2m canopy; **Record Level:** institutionCode: LUNZ; datasetName: Hope River Forest Fragmentation Project; basisOfRecord: PreservedSpecimen
- **Type status:** Other material. **Occurrence:** catalogNumber: #0397-024; recordedBy: Raphael K. Didham; individualCount: 1; sex: male; lifeStage: adult; **Taxon:** scientificName: Stenosagola newtoni Park & Carlton, 2013; **Location:** country: New Zealand; verbatimLocality: Umbrella (0.02ha); verbatimLatitude: 42.38S; verbatimLongitude: 172.24E; verbatimCoordinateSystem: degrees minutes; **Identification:** identifiedBy: Stephen E. Thorpe; dateIdentified: 2014; identificationReferences: Park, J.-S.; Carlton, C.E. 2013: A revision of the New Zealand genus Stenosagola Broun, 1921 (Coleoptera: Staphylinidae: Pselaphinae: Faronitae). Coleopterists bulletin, 67(3): 335-359. doi: 10.1649/0010-065X-67.3.335; identificationRemarks: Aedeagus examined. Mirror image of fig. 12d in Park & Carlton (2013); **Event:** eventID: #0397; samplingProtocol: FIT; samplingEffort: 12 days; eventDate: 2000-12-18; habitat: forest 2m ground; **Record Level:** institutionCode: LUNZ; datasetName: Hope River Forest Fragmentation Project; basisOfRecord: PreservedSpecimen
- **Type status:** Other material. **Occurrence:** catalogNumber: #0392[-001]; recordedBy: Raphael K. Didham; individualCount: 1; sex: male; lifeStage: adult; **Taxon:** scientificName: Stenosagola newtoni Park & Carlton, 2013; **Location:** country: New Zealand; verbatimLocality: Windy Point (0.04ha); verbatimLatitude: 42.39S; verbatimLongitude: 172.23E; verbatimCoordinateSystem: degrees minutes; **Identification:** identifiedBy: Stephen E. Thorpe; dateIdentified: 2014; identificationReferences: Park, J.-S.; Carlton, C.E. 2013: A revision of the New Zealand genus Stenosagola Broun, 1921 (Coleoptera: Staphylinidae: Pselaphinae: Faronitae). Coleopterists bulletin, 67(3): 335-359. doi: 10.1649/0010-065X-67.3.335; identificationRemarks: Aedeagus examined. Mirror image of fig. 12d in Park & Carlton (2013); **Event:** eventID: #392; samplingProtocol: FIT; samplingEffort: 16 days; eventDate: 2000-12-18; habitat: forest 8m canopy; **Record Level:** institutionCode: LUNZ; datasetName: Hope River Forest Fragmentation Project; basisOfRecord: PreservedSpecimen
- **Type status:** Other material. **Occurrence:** catalogNumber: #389-003; recordedBy: Raphael K. Didham; individualCount: 1; sex: male; lifeStage: adult; **Taxon:** scientificName: Stenosagola newtoni Park & Carlton, 2013; **Location:** country: New Zealand; verbatimLocality: Windy Point (0.04ha); verbatimLatitude: 42.39S; verbatimLongitude: 172.23E; verbatimCoordinateSystem: degrees minutes; **Identification:** identifiedBy: Stephen E. Thorpe; dateIdentified: 2014; identificationReferences: Park, J.-S.; Carlton, C.E. 2013: A revision of the New Zealand genus Stenosagola Broun, 1921 (Coleoptera: Staphylinidae: Pselaphinae: Faronitae). Coleopterists bulletin, 67(3): 335-359. doi: 10.1649/0010-065X-67.3.335; identificationRemarks: Aedeagus not examined; **Event:** eventID: #389; samplingProtocol: FIT; samplingEffort: 16 days; eventDate: 2000-12-18; habitat: forest 0m canopy; **Record Level:** institutionCode: LUNZ; datasetName: Hope River Forest Fragmentation Project; basisOfRecord: PreservedSpecimen
- **Type status:** Other material. **Occurrence:** catalogNumber: #0388-001; recordedBy: Raphael K. Didham; individualCount: 1; sex: male; lifeStage: adult; **Taxon:** scientificName: Stenosagola newtoni Park & Carlton, 2013; **Location:** country: New Zealand; verbatimLocality: Windy Point (0.04ha); verbatimLatitude: 42.39S; verbatimLongitude: 172.23E; verbatimCoordinateSystem: degrees minutes; **Identification:** identifiedBy: Stephen E. Thorpe; dateIdentified: 2014; identificationReferences: Park, J.-S.; Carlton, C.E. 2013: A revision of the New Zealand genus Stenosagola Broun, 1921 (Coleoptera: Staphylinidae: Pselaphinae: Faronitae). Coleopterists bulletin, 67(3): 335-359. doi: 10.1649/0010-065X-67.3.335; identificationRemarks: Aedeagus not examined; **Event:** eventID: #388; samplingProtocol: FIT; samplingEffort: 16 days; eventDate: 2000-12-18; habitat: forest 8m ground; **Record Level:** institutionCode: LUNZ; datasetName: Hope River Forest Fragmentation Project; basisOfRecord: PreservedSpecimen

#### Diagnosis

**Figure 1.**
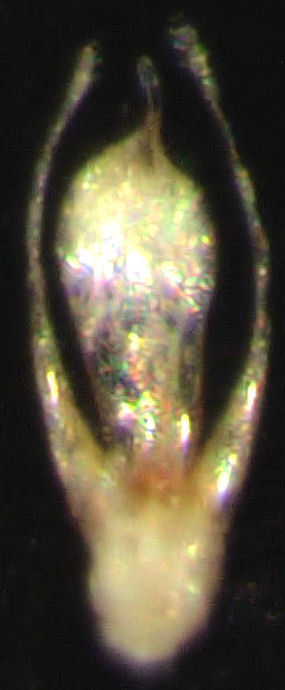
Aedeagus (dorsal view) of specimen #339[-001].

**Figure 2.**
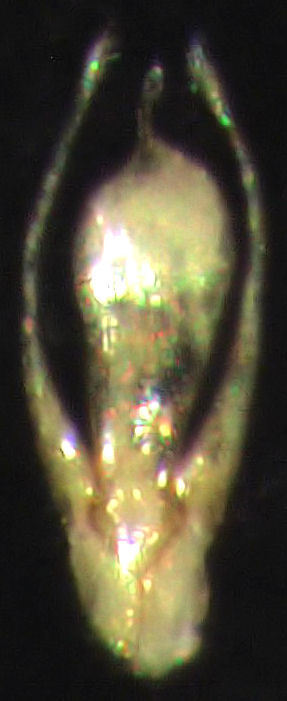
Aedeagus (ventral view) of specimen #339[-001].

*Stenosagola newtoni* can only be recognised by the aedeagus (Fig. 1, Fig. 2). The shape of the median lobe is diagnostic. It is broad and flattened, with a slender apical lobe. The apical lobe is usually curved to the left in dorsal view (Fig. 1). The apical lobe is closer to the right hand margin of the main body of the median lobe than it is to the left hand margin. The left hand margin is more convex (in the apical half) than the right hand margin.

#### Distribution

South Island of New Zealand.

## Discussion

Aedeagal antisymmetry is a phenomenon whereby there can be mirror image variation in male genitalia (Schilthuizen 2013). All the aedeagi examined for the present note are the mirror image of fig. 12d in Park and Carlton (2013). However, the type specimens of *Stenosagola newtoni* in fact have identical aedeagal symmetry to the HRFFP material, and the mirror image of the aedeagus was inadvertently illustrated in fig. 12d (J.-S. Park pers. comm., 2014).

The following 8 specimens are from the HRFFP samples, but were not used in the analysis by Ewers et al. (2007): #284-007, #339[-001], #378[-001], #389-003, #392[-001], #482[-001], #1049[-001], and #1646[-001]. They have all been newly identified herein as *Stenosagola newtoni*. Of the 42 specimens of morphospecies sp. 0471 which were used in the analysis, 41 are newly identified herein as *Stenosagola newtoni*, but one specimen (#0934-008) is herein determined to belong to the Euplectitae.

## Supplementary Material

XML Treatment for Stenosagola newtoni
